# Comparison of dmft and behavior rating scores between children with systemic disease and healthy children at the first dental visit

**DOI:** 10.1186/s12903-024-04285-8

**Published:** 2024-05-10

**Authors:** Gizem Erbas Unverdi, Beste Ozgur, Hamdi Cem Gungor, Paul S. Casamassimo

**Affiliations:** 1 Department of Pediatric Dentistry, Faculty of Dentistry, Hacettepe University, 06100 Sihhiye, Ankara, Turkey; 2 Division of Pediatric Dentistry, Department of Developmental Sciences, Marquette University School of Dentistry, Milwaukee, WI USA; 3 Division of Pediatric Dentistry, Ohio State University (Research Center), Columbus, OH USA

**Keywords:** Oral health, Children, Chronic disease, Cooperation

## Abstract

**Purpose:**

To evaluate and compare oral health and behavior scores at the first dental visit and dental treatment need using general anesthesia/sedation **(GA/S)** of children with systemic diseases (**SD**) and healthy children.

**Methods:**

Data were obtained from healthy children (*n* = 87) and children with SD (*n* = 79), aged 4 to 6 years, presenting to a hospital dental clinic for a first dental examination. The total number of decayed, missing and filled teeth **(dmft)**, dental behavior score using Frankl Scale, and dental treatment need using GA/S were recorded. Chi-square / Fisher’s exact test and Mann–Whitney U tests were used for statistical analyses.

**Results:**

The patients with SD were diagnosed with cardiac disease (61%), renal disease (9%), and pediatric cancers (30%). The median dmft values of the SD group (3.00) were significantly lower than those of healthy children (5.00) (*p* = 0.02) and healthy children exhibited significantly more positive behavior (90.8%) than children with SD (73.4%) (*p* = 0.002). The number of patients needing GA/S for dental treatment did not differ significantly between the two groups (*p* = 0.185). There was no relationship between dental treatment need with GA/S and dental behavior scores of the patients (*p* = 0.05). A statistically significant relationship was found between the patients’ dmft scores and the need for dental treatment using GA/S; and the cut-off value was found to be dmft > 4 for the overall comparisons.

**Conclusion:**

The presence of chronic disease in children appeared to affect the cooperation negatively at the first dental visit compared to healthy controls, however, it did not affect the oral health negatively. Having a negative behavior score or SD did not necessitate the use of GA/S for dental treatment.

## Introduction

Challenges in managing children’s behavior are one of the most frequent reasons for referral to a pediatric dentist. The behavior of a child during dental treatments can be influenced by numerous factors, such as age, the parents’ level of education, the child’s oral health status, previous negative dental treatment experiences, traumatic and/or frequent medical experiences, and dental pain [1, 2]. The way in which a child initially deals with stressors is related to personal experiences, as well as the experiences of parents, family, and friends [3]. The child’s first dental experience and impressions play a pivotal role in establishing a dependable relationship between the child and the dental practitioner [4]. Moreover, the first dental appointment allows the dentist to predict the child’s behavior during the dental procedure, enabling the dentist to apply proper techniques effectively [1, 3, 4]. 

Systemic disease (**SD**) in children may affect oral and dental health through different mechanisms [5]. Oral health of children with SD can be at risk from several factors, including long-term use of sugar-containing oral medications; the need for a diet rich in carbohydrates or the use of medically necessary high calorie supplements (e.g., for weight gaining). Also, the side effects of medications (e.g., xerostomia) should be reviewed as an impact on caries and periodontal problems [6, 7]. Furthermore, the likelihood of having untreated dental problems increases with the severity of their health conditions [6, 8]. Minimizing the risk of developing oral disease is a fundamental part of comprehensive oral and dental health care for children with SD [6, 7]. 

A child with SD may also have dental anxiety/fear which further compounds the problem when dental treatment is needed [6]. Behavior management techniques and communicative approaches are used to introduce the dental setting to children, but behavior guidance for a patient with SD can also be challenging [9]. SD has been shown to be important in the development of dental anxiety and/or fear in children, particularly with disorders that require frequent medical visits such as attention deficit hyperactivity disorder **(ADHD)**, pediatric cancers, cleft lip/cleft palate, and congenital heart disease **(CHD)** [10–13]. Dental anxiety/fear and having a systemic disease can both cause irregular dental attendance, remarkable intolerance, and limitation during dental treatment which may later become factors for poor dental health [11, 14–16]. Colares and Richman [2] have related general health problems and previous hospitalization with the behavior of children in dental settings. Dentally anxious children may present more behavior management problems [17], elevated levels of dental caries [2] and have more experience of general anesthesia/sedation **(GA/S)** for dental treatment [18]. Uncooperative behavior may necessitate the use of pharmacological behavior-modifying techniques such as sedation or general anesthesia to achieve better quality dental treatment in a non-stressful environment [9]. 

The assessment of dental behavior in children with SD who experience anxiety/fear is essential to perform quality clinical dental procedures. Understanding how these patients respond to clinical dental procedures and the factors involved can guide their management by clinicians and facilitate their access to health services [19]. The reluctance of dentists to see children with SD, or their lack of comfort in treating these children, adds to the likelihood that these children will not receive necessary beneficial preventive and treatment services [6, 20]. This is especially true for school-aged children who are likely to benefit most from early dental visits [21]. A better understanding of the potential challenges that school-aged children with SD would present to dentists compared to healthy children is needed. Hence, the research question of the present study was “Is there a significant difference in dmft scores and behavior rating scores at the first dental visit, and need for GA/S for dental treatment in children with SD compared to healthy controls?”.

## Materials and methods

This retrospective case-control study was approved by the Institutional Review Board **(IRB)** of Nationwide Children’s Hospital **(NCH)**, Columbus, Ohio (IRB16-01258). The data of patients presenting to NCH dental clinic for their first dental examination/hygiene appointment between January 1, 2012, and January 1, 2017, were collected.

### Subjects

For the study group (SD), the inclusion criteria were listed as: (1) patients 4 to 6 years of age with a primary diagnosis of a systemic disease (pediatric cancers, cystic fibrosis, hydrocephalus, spina bifida, heart, kidney diseases, etc.) that require at least one surgical medical procedure; (2) patients with no history of dental procedures elsewhere were included. For the comparison group, healthy patients, ages 4 to 6 years; no diagnoses of systemic medical conditions and no history of earlier dental procedures were included. For both groups, patients who received only a dental examination or preventive treatment during their first dental visit in the pediatric dentistry clinic were included in the study. The patients with the following criteria were excluded from the study:

- Patients with no recorded dental behavior score on their charts.
- Patients who received surgical/restorative dental treatment at the first dental visit or presented with dental pain/dental traumatic injury.
- Patients with diagnosed behavioral or cognitive disorders, syndromes, or those using medications that may affect their behavior (e.g., mood-stabilizing/mood-altering medications, medications for ADHD, etc.)
- In the study group, patients who underwent medical surgical procedures after their first dental visits.

All dental treatments were provided by the first- or second-year residents enrolled in the advanced training program in pediatric dentistry. Since this was a retrospective study, no kappa scores were looked for among the examiners. However, all residents underwent the same comprehensive training program to be able to assess and decide on a patient’s dental behavior score and the dental treatment need using GA/S. If the resident had any difficulty in deciding on the GA/S need, they would consult the supervisors and come to a decision with consensus. The sample size calculation was based on the results of a pilot study involving 16 randomly selected children for each group. Dental behavior scored by Frankl scale was considered as the main variable. “Negative” and “definitely negative” behavior were observed in 25% of children with SD and in 6.3% of healthy children in the control group. G power 3.1.9.2 software (Franz Faul, Kiel, University, Germany, RID: SCR_013726) was used, considering a power of 82% (1-β equals 0.82), and an error rate of α equals 0.05. A required sample size of 62 was calculated for each group. The main goal of the pilot study was only to decide the sample size, and the patients involved in the pilot study were not included in the main study.

The data were collected from the Electronic Patient Information Chart (EPIC, Epic Systems Corporation, Verona, WI, USA) of NCH by one trained pediatric dentist (GEU). For the calibration purposes, the pediatric dentist underwent a comprehensive training program, ensuring a solid understanding of data collection procedures and evaluation criteria. Practical training was provided using sample data, and the pediatric dentist received hands-on guidance from experienced supervisors during simulated data collection. To enhance consistency and accuracy, test cases were introduced to confirm the pediatric dentist’s data collection and evaluation skills. Additionally, an independent evaluator reviewed a subset of collected data to confirm the accuracy and precision of the process. There was also another experienced pediatric dentist and a supervisor for making a consensus when the pediatric dentist was not sure about the collected data.

**Fig. 1:**
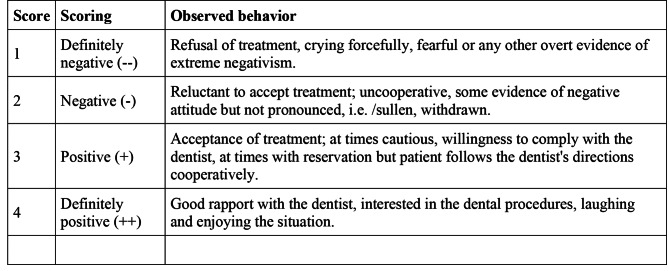
Frankl behavior rating scale used in the study for assessing the behavior of children

A total of 347 patients who were between the ages of 4 and 6 years and had their first dental visit at the selected dental clinic were found. They received no previous dental procedures elsewhere. The charts of these patients were examined by a pediatric dentist (GEU) and a total of 166 patients, comprising healthy controls and children with SD were included in the study. The remaining 181 patients were excluded based on the exclusion criteria. Information regarding age, gender, behavior during at the first dental visit, primary and additional diagnoses, total number of decayed, missing and filled teeth (dmft), GA/S need for dental treatment, behavior score assessed with Frankl Behavior Rating Scale [22] (Fig. 1) (i.e., definitively negative [− −]; negative [−]; positive [+] and definitively positive [++]) were entered into Excel Spreadsheet Software (Microsoft Inc., Redmond, WA, USA).

### Statistical analyses

The Kolmogorov-Smirnov and Shapiro Wilk tests were used to test whether data were normally distributed. Homogeneities of variance were tested by Levene’s test. Student’s t-test or Mann-Whitney U test was used to compare the study and control groups for continuous variables. The Chi-Square test or Fisher’s exact test (where the chi-squared test was not appropriate if the expected values in any of the cells of a contingency table were below 5, or below 10 if there was only one degree of freedom) were used to determine a significant difference between the expected and observed frequencies. The continuous variables within the SD group were compared using Kruskal Wallis test. The diagnostic power of dmft value for GA/S need for dental treatment was investigated by ROC analysis. The area under the ROC curve gives an estimate of the overall accuracy of each group. An area of 0.50 implies that the variable adds no information. The areas under the ROC curves and 95% confidence intervals for responsible variables were calculated as described by Hanley and McNeil and a cutoff value was estimated using the index of Youden. Frequencies (percentages), mean ± standard deviation and median (Q1-Q3) were given as descriptive statistics. Statistical analyses were performed using IBM® SPSS® 23.0 for Windows (IBM Corp., Armonk, NY, USA), where *p* < 0.05 was considered statistically significant.

## Results

The data of 79 children with SD and 87 healthy children were analyzed. After checking regarding the inclusion and exclusion criteria, only those with pediatric cancer, heart and renal disease remained among the patients who could be included into the study. In SD group, 61% of the patients had heart disease, while 30% and 9% had cancer and renal disease, respectively. The distribution of age, gender and dmft values of the groups and subgroups of SD is presented in Table 1. The male-to-female ratios in the SD and control group were 1.55 and 1.02, respectively. The control group had a median age of 4.82 years, while it was 4.77 years for the SD group. No significant difference was found between the groups for age and gender (*p* = 0.62 and *p* = 0.18, respectively) (Table 1).

**Table 1:** The distribution and comparison of gender, age and dmft values of the groups; and the distribution of patients and dmft within the systemic disease group*

	Control Group	Systemic Disease Group	
	n (%)**	n (%)**	**p**†
**Female** **Male**	43 (49%) 44 (51%)	31 (39%) 48 (61%)	0.18
**Total**	87 (100%)	79 (100%)	
	**Median (Q1-Q3) - Mean ± SD**		**p** ^‡^
**Age**	4.82 (4.41–5.30) – 4.91 ± 0.56	4.77 (4.35–5.29) – 4.87 ± 0.57	0.62
**Dmft value**	5.00 (0.0–9.0) – 5.44 ± 4.47	3.00 (0.0–7.0) – 3.88 ± 3.67	**0.02**
	**Systemic Disease Group (dmft)**		
Disease (n (%)**)	**Median (Q1-Q3) - Mean ± SD**		**p** ^§^
**Cardiac (**48 (61%)**)** **Cancer (**24 (30%)**)** **Renal (**7 (9%)**)**	5.00 (1.25-8) – 4.81 ± 3.74 ^b^ 0.00 (0–4) – 2.00 ± 2.83 ^a^ 2.00 (0–6) – 3.14 ± 3.44 ^a,b^		**0.008**

* Abbreviations used in this table: n: number; Q1-Q3: first and third interquartile range; SD = standard deviation; dmft = decayed, missing, filled teeth

** Column percentage

† *P*-value using Chi-square test

‡ *P*-value using Mann-Whitney U test

§ *P*-value using Kruskal Wallis test for dmft value

The median dmft of patients with SD and the control group were 3.00 and 5.00, respectively. SD group’s dmft was significantly lower than the control group (*p* = 0.02). Within the SD group, the median dmft values among systemic diseases were 5.00, 0.00 and 2.00 for cardiac disease, pediatric cancer, and renal disease, respectively. Patients with cardiac disease had significantly higher median dmft values than patients with pediatric cancer (*p* = 0.008) (Table 1). Twenty-eight patients in the SD group (35.4%) were caries-free/negative (dmft = 0) compared to 18 patients (20.7%) in the control group. The caries status differed significantly between the groups (*p* = 0.03) (Table 2).

**Table 2:** Distribution and comparison of caries prevalence, Frankl scale values and the dental treatment need with general anesthesia/sedation of the patients in the groups*

		Group			
		Control *n* (%**)	Systemic *n* (%**)	Total *n*	*p*
**Caries** **Prevalance**	Positive (dmft > 0) Negative (dmft = 0)	69 (79.3)	51 (64.6)	120	**0.03** ^**§**^
		18 (20.7)	28 (35.4)	46	
**Frankl scale**	Score 1 (D.Negative)	0 (0.0)	10 (12.7)	10	**0.002**†
	Score 2 (Negative)	8 (9.2)	11 (13.9)	19	
	Score 3 (Positive)	28 (32.2)	24 (30.4)	52	
	Score 4 (D.Positive)	51 (58.6)	34 (43.0)	85	
**Total**		87	79	166	
**General Anesthesia/Sedation**‡	Yes	36 (52.2)	34 (64.2)	70	0.18^**§**^
	No	33 (47.8)	19 (35.8)	52	
**Total (n)**		69	53	122	

* Abbreviations used in this table: dmft = decayed, missing, filled teeth; D.Negative = definitely negative; D.Positive = definitely positive

**Column percentage

^**§**^*P*-value using Chi-squared analysis

† *P*-value using Fisher’s exact test

‡ Only the patients that needed a dental treatment were included (Patients that had dmft > 0 or even if dmft = 0, they needed scaling)

Table 2 shows the caries prevalence and Frankl scores in the groups, and the distribution of patients who needed GA/S for dental treatment. There was a significant difference between the groups in relation to dental behavior (*p* = 0.002). None of the patients in the control group showed “definitely negative” behavior. Of the patients with SD, 26.6% presented uncooperative behavior (either “negative” or “definitely negative”), while 43% of them had “definitely positive” behavior. In the control group, 9.2% of the patients were uncooperative, and 58.6% exhibited “definitely positive” behavior. The percentage of patients who needed GA/S for dental treatment did not differ significantly in SD (48.6%) and control groups (51.4%) (*p* = 0.18).

For the statistical analysis in Table 3, behavior scores were dichotomized into one “positive” and one “negative” score by converting two positive/negative scores into one negative and positive score. There was no significant difference between dental behavior scores of patients and GA/S need for both intragroup and overall comparisons (*p* = 0.45 for control, *p* = 0.13 for SD group, *p* = 0.05 for overall).

**Table 3:** Comparison of patients’ dental behavior scores and treatment needs with GA/Sedation

			Frankl Score Negative*	Frankl Score Positive*	Total	P**
			**n (%)**†	**n (%)**†	**n**	
**Treatment with GA/Sedation** ^‡^	**Control group**	**Yes**	4 (11.1)	32 (88.9)	36	0.675
		**No**	2 (6.1)	31 (93.9)	33	
	**Systemic group**	**Yes**	12 (35.3)	22 (64.8)	34	0.205
		**No**	3 (15.8)	16 (84.2)	19	
	**Total**	**Yes**	16 (22.9)	54 (77.1)	70	0.088
		**No**	5 (9.6)	47 (90.4)	52	

* Behavior scores were dichotomized into one “positive” and one “negative” score by converting two positive/negative scores into one negative and positive score ^19^

** *P*-value using Fisher’s Exact test

† Row percentage

‡ Only the patients that needed a dental treatment were included (Patients that had dmft > 0 or even if dmft = 0, they needed scaling)

The comparison between patients’ dmft values and dental treatment need using GA/S was shown in Table 4. A statistically significant relationship was found between these variables for both intragroup and the overall comparisons (*p* < 0.001) (Table 5). The dmft cut-off value for the SD group was dmft > 5. However, for the control group and overall comparisons, the cut-off value was dmft > 4 (Table 5).

**Table 4:** Comparison of patients’ dmft values and dental treatment needs with GA/Sedation*

			dmft			
			Mean ± SD		Median (Q1-Q3)	P**
**Treatment** **with GA/Sedation**	**Control group**	**Yes**	9.25 ± 3.15		9.0 (8.0–11.0)	**< 0.001**
		**No**	2.74 ± 3.09		2.0 (0.0–4.0)	
	**Systemic group**	**Yes**	6.70 ± 3.07		7.0 (6.0–8.0)	**< 0.001**
		**No**	1.65 ± 2.32		0.0 (0.0–3.0)	
	**Total**	**Yes**	8.01 ± 3.34		8.0 (0.0–10.0)	**< 0.001**
		**No**	2.24 ± 2.80		1.0 (0.0–4.0)	

* Abbreviations used in this table: dmft = decayed, missing, filled teeth; SD = standard deviation; GA = general anesthesia

** *P*-value using Mann Whitney U test

**Table 5:** The cut off values of dmft for the groups that identify the dental treatment need with general anesthesia/sedation*

	Area	Std. Error	95% Conf. Interval		P**	CutOff value (dmft)	Sensitivity	Specificity
			Lower	Upper				
**Control**	0.922	0.029	0.864	0.980	**< 0.001**	> 4	94.44	78.43
**Systemic**	0.894	0.038	0.820	0.969	**< 0.001**	> 5	76.47	88.37
**Total**	0.900	0.025	0.851	0.949	**< 0.001**	> 4	87.14	80.85

* Abbreviations used in this table: dmft = decayed, missing, filled teeth; Std. Error = standard error

** Cut of values calculated by using According to Youden Index

## Discussion

Studies have investigated dental health status and/or behavior of patients with one specific systemic disease such as autism [23, 24], cerebral palsy [19], cardiovascular disease [25], and Down syndrome [26]. The present study is the first to compare children with systemic disease to healthy children with respect to dental behavior at first dental visit, dental caries, and GA/S need for dental treatment.

Several studies have compared dental caries status of children with CHD to that of healthy children [14, 25, 27–30]. Most of the studies reported no significant difference in relation to caries prevalence [14, 25, 29, 30]. A few studies [27, 28] found lower mean dmft values in healthy patient groups when compared to the study groups [25, 27–30]. In those earlier studies, mean dmft values of patients with CHD ranged between 1.03 and 3.7 [14, 25, 27, 28], which was lower than those of patients with CHD in the present study (4.81). However, the age range was wider (1–16 years old) in latter mentioned studies [14, 27, 28, 30]. As an exception, in one recent study, Sarac et al [31]. reported the mean dmft value of children with CHD in primary dentition as 5.4, which was higher than the latter mentioned studies and close to the results of the present study. A study with renal dialysis patients reported that almost all patients (99.4%) were caries positive [32]. Kowlessar et al.[33] assessed the oral health of pediatric oncology patients and reported the prevalence of dental caries in the primary dentition as 54.3%. Furthermore, these children exhibited a dental caries experience ranging from 2.28 (mean dmft) to 3.2 (mean dft), potentially attributed to their exposure to chemoradiation therapy [33, 34]. Those findings were higher than the mean dmft value of the children with pediatric cancer (2.00) in the present study. Moreover, the current study found that the dmft values of children in the SD group were significantly lower than that of healthy patients. This is in line with the findings of Ronis et al. [35], who noted better oral health in special needs preschoolers than healthy children. Hence a presumption of poorer oral health and challenges in children with SD may be unfounded. The SD group in the current study represents a rare population with a high severity of disease, which require health care as an integral part of their life. Better caries status maybe because of their parents who have reached to an understanding of the significance of oral health through the interactions with their children’s healthcare providers. On the other hand, it should be noted that within the SD group of the present study, the percentages of subgroups were not equal (61% for cardiac, 30% for cancer, 9% for renal disease), which could be misleading about the overall average of dmft value in SD group. This non-homogeneity was expected since the number of each SD subgroup was not predetermined beforehand. It was limited by the number of patients presenting during the study period. Accordingly, the dmft value among children with cardiac disease were the highest and was similar to the healthy children. It has been reported that children with cancer are also at increased risk of dental caries due to malnutrition, xerostomia and the use of high-sugar medicines [36] The children with childhood cancer in the present study had the lowest dmft score in the SD group. However, it should be taken into account that due to the lower dmft scores in children with pediatric cancer in the current study, the overall mean dmft value in the SD group might have decreased.

The strategies for managing stress and anxiety within a dental setting differ significantly and have distinct attributes during childhood and adolescence. Following the first encounter, these coping mechanisms become ingrained, and this experience may affect the behavior of a child during future dental treatments [3]. Therefore, in the present study, children who had their first dental appointment were selected for evaluating the dental behavior. Frankl Behavior Rating Scale is a reliable and functional method that quantifies behavior into four categories [22]. Healthy children showed significantly more positive dental behavior than patients with SD. No previous research was found that compared behavior of the children with systemic diseases during the first dental visit. A few studies about the aspects of dental anxiety or fear of these patients to dental treatments exist [11, 13, 15, 31]. Pediatric cardiology patients were reported to have significantly increased levels of dental anxiety compared to healthy children and the history of their medical interventions or overnight hospital admissions were likely to be the contributory factors [11]. Increased anxiety, stress, and impaired dental behavior were reported in renal dialysis patients by Dumitrescu et al. [32] The study by Galili et al. [37] showed significantly less dental anxiety in chronic hemodialysis patients compared to the healthy group. On the other hand, in a cross-sectional study comparing childhood cancer survivors with healthy children aged 6 to 14 years old, Wogelius et al. [13] reported that having cancer and cancer treatment during childhood did not seem to increase the risk of dental anxiety.

One of the aims in this study was to compare the presenting characteristics of young children, with and without systemic conditions, during their first dental visits. When terms like special needs, systemic illness, or specific condition diagnoses (e.g., Down Syndrome) are reported by caregivers to dentists for scheduling a first visit, barriers may be put up that may prevent a child from being seen. This study suggests that, at an early age, children may exhibit similar characteristics whether they have a health issue or not. When choosing a suitable behavioral management technique, clinicians should assess the patient’s oral health status, cognitive, emotional, and physical development, the complexity of the required dental treatment and the parental characteristics [38]. Based on the results of this study, that approach seems advisable as an initial strategy for all young children. This study looked at children who were seen in an ambulatory setting, duplicating community-based engagement rather than in-house dental consultation on an admitted child with a systemic illness, thus reflecting what a dentist might expect to experience.

Behavior of school-aged children at the first visit often directs the choice of advanced behavior guidance. Pharmacological techniques, such as GA/S, are commonly used to deliver extensive dental treatment to patients with anxiety, physical disability, cognitive disorders, or challenging behavior, many of whom are children with special needs [38, 39]. Specifically, uncooperative behavior has been identified as a significant indicator for GA/S, not only in studies with special needs patients, but also in studies with healthy children [39–41]. The review of Helsinki Public Dental Service in Finland found that extreme uncooperative behavior was the main reason for a considerable number of patients (65%) to undergo GA for dental treatment, followed by dental phobia (37%) and excessive treatment need (26%) [40, 42]. In the current study, although, a significantly higher portion of uncooperative behavior was seen in the group with SD, GA/S need for dental treatment was similar for both groups. Karim et al. [42]. reported that in 369 patients undergoing GA for dental treatment, 43% had medical problems, similar to the percentage (43.4%) in the present study [42]. Apart from these findings, the present study showed that dmft values were related to GA/S need for dental treatment. The probability of dental treatment using GA/S increased in the control group, when dmft value was greater than 4. However, in SD group, it increased when dmft value was greater than 5.

The limitations of the present retrospective case-control study could be stated as follows: This is a single-center study, whose sample size may not be representative of all children with the systemic diseases evaluated. Furthermore, it was not possible to compare the study group with the matched controls, since there were no other patients who met the inclusion/exclusion criteria and could be included in the study. More multicenter studies with larger sample sizes and matched control groups are required. Another limitation was that the information of patients such as socio-demographics, oral hygiene and/or dietary habits which may be the determinants of caries status and severity was not obtained. Dental exams were made by different pediatric dentists. This might have contributed to a variability in assessing the children’s dental behavior and the decision of dental treatment need with GA/S.

A consistent set of criteria for caries and referral for GA/S, the training for caries assessment and behavior rating, a limited number of dentist-providers, and an electronic health record that focuses on choices can be stated as the study’s strengths. Additionally, this study simulated a community-based provider engagement rather than an in-patient consultation, so the results more likely reflect the behavior of children with SD seeking dental care in a community setting. The results may be useful for community placement of children, especially those distant from major medical centers.

Early intervention has been shown to improve outcomes [21]. This study findings suggest that irrespective of the age and the systemic disease status of a child, behavior and caries status could be considered during treatment planning which often require general anesthesia or sedation. In an aim to optimize preventive and treatment options for the pediatric patients and their families, the clinicians should be prepared to address these two factors along with timely referral in some cases.

## Conclusions

This study has shown that the presence of SD in children appeared to affect the cooperation negatively at the first dental visit compared to healthy controls. In children, the presence of SD was not found to have a negative effect on oral health in comparison with healthy controls. Having SD or a negative behavior score in children did not necessitate the use of GA/S for dental treatment.

## Data Availability

The datasets used and/or analyzed during the current study available from the corresponding author on reasonable request.

## Abbreviations

GA/S: General anesthesia/Sedation
SD: Systemic diseases
dmft: decayed, missing and filled teeth
ADHD: Attention deficit hyperactivity disorder
CHD: Congenital heart disease
IRB: Institutional Review Board
NCH: Nationwide Children’s Hospital
ROC: Receiver Operating Characteristic
